# Intergenerational Transmission of Resilience? Sense of Coherence Is Associated between Lithuanian Survivors of Political Violence and Their Adult Offspring

**DOI:** 10.3389/fpsyg.2017.01677

**Published:** 2017-09-26

**Authors:** Evaldas Kazlauskas, Danute Gailiene, Ieva Vaskeliene, Monika Skeryte-Kazlauskiene

**Affiliations:** Department of Clinical and Organizational Psychology, Vilnius University, Vilnius, Lithuania

**Keywords:** trauma, post-traumatic stress, sense of coherence, intergenerational, resilience, political oppression, Lithuania

## Abstract

Little is known about intergeneration effects on mental health in the families of survivors of political oppression of communist regime in Central and Eastern Europe. We aimed to explore post-traumatic stress in the second generation of the Lithuanian survivors of political violence, and analyze links between parental and adult offsprings’ sense of coherence in the families exposed to political violence during the oppressive communist regime in Lithuania. A total of 110 matched pairs of communist regime political violence survivors (mean age = 73.22 years) and their adult offspring (mean age = 44.65 years) participated in this study. Life-time traumatic experiences and sense of coherence were measured in both parents and their offspring. Post-traumatic stress symptoms were assessed in the second generation of survivors. We found a high vulnerability in the second generation of the Lithuanian families of political violence survivors, with a 29% of probable PTSD in the second generation based on self-report measures. A significant positive correlation between parental and adult offsprings’ sense of coherence was found. Post-traumatic stress symptoms were associated negatively with a sense of coherence in the second generation. Our study indicates the links between parental and the second generation’s sense of coherence in the families of survivors of political violence. The study raises broader questions about the intergenerational aspects of resilience. Further studies are needed to explore the links between parental and child sense of coherence in other samples.

## Introduction

Inspired by research of the effects of the Holocaust on the second generation, the field of intergenerational trauma has progressed significantly during the last few decades. Intergenerational trauma studies, once originated within Holocaust survivors’ second generation studies, are now expanding into many more areas. There are findings available from studies on intergenerational effects in families of war veterans (Dekel and Goldblatt, 2008; Dias et al., 2014), war prisoners (Zerach and Solomon, 2016), political oppression (Vaskeliene, 2012; Javakhishvili, 2014), refugees (van Ee et al., 2012) and others, exploring the effects of parental trauma on the second or the third generation. However, there are still debates going on about intergenerational trauma among mental health researchers and professionals. There are studies revealing that offspring of Holocaust survivors can adapt quite well (van IJzendoorn et al., 2003). A meta-analysis by van IJzendoorn et al. (2003) has shown that effects of trauma on the second generation do not necessary result in severe clinical outcomes. On the other hand, there is considerable evidence that parental trauma can have significant effects on offspring (Bowers and Yehuda, 2016). This ongoing scientific debate is an encouragement to conduct further research and identify who are the most vulnerable among the second generation (Danieli et al., 2016). Considering the mixed research results about the intergenerational effects of parental trauma (van IJzendoorn et al., 2003; Danieli et al., 2016), further studies of intergenerational trauma transmission and resilience are necessary.

Trauma-related parental stress can produce neuroendocrine, epigenetic, and neuroanatomical changes in offspring as evidenced by genetic and neuroscience research (Bowers and Yehuda, 2016; Yehuda et al., 2016). The current trends in intergenerational trauma research are directed toward the need to understand the underlying mechanisms of trauma transmission (Bowers and Yehuda, 2016). Several pathways of trauma transmission have been conceptualized recently, including parental stress-related, environmental, parenting, genetic, epigenetic factors (Binder et al., 2008), and attachment-related factors (Bar-On et al., 1998). There is growing support from genetic and epigenetic studies, especially associated with the gene FKBP5, revealing the biological mechanisms of trauma transmission (Binder et al., 2008; Yehuda et al., 2016). In addition psychological factors, such as parenting, perceived parental burden, attachment and family communication are also relevant for an understanding of trauma transmission in families (Wiseman et al., 2002; Letzter-Pouw et al., 2014; Shrira, 2016).

### Long-Term Effects of Communist Oppression in Europe

In the 1940s the Second World War related atrocities significantly affected the population of Eastern and Central European countries. Moreover, many families suffered from the oppressive communist regime in these countries. The forced displacement of a large proportion of the population to very remote regions of Siberia (Gailiene and Kazlauskas, 2005), political imprisonments, the abuse of psychiatry (van Voren, 2010), and other forms of repression were prevalent in the middle of the 20th century in these countries, and lasted until the collapse of the Soviet Union in the 1990’s. Only after the political changes, studies of long-term psychological effects of political oppression began. Our knowledge about long-term effects on the mental health of survivors is limited due to the lack of studies in the region. However, research has shown long-term psychological effects on survivors of communist political violence with high rates of PTSD and other mental disorders from Romania (Bichescu et al., 2005), East Germany (Bauer et al., 1993; Maercker and Schützwohl, 1997), and Lithuania (Gailiene and Kazlauskas, 2005; Kazlauskas, 2006).

There are only several studies available that explored the intergenerational effects of Soviet era communist political oppression in Eastern and Central Europe. Research on the second or third generation of survivors from Russia (Baker and Gippenreiter, 1998), Georgia (Javakhishvili, 2014), and Lithuania (Vaskeliene, 2012) reported the specific communication patterns in families marked with secrecy surrounding the political violence-related trauma in the families. The lack of studies sometimes is explained by the controversies and mixed opinions on communist regime-related traumatization in Europe (Kattago, 2009; Gailiene, 2015), but it is also largely related with the oppression of psychology as a profession in these countries during the Soviet era (Kazlauskas and Zelviene, 2016), and the lack of resources after the political change.

### Theoretical Background of This Study

With evidence that the second generation can be affected by parental trauma, there is a growing number of studies aiming to analyze intergenerational resilience. Several important factors of intergenerational resilience have been identified recently, such as parenting style (Field et al., 2013), and communication in families as a protective factor in the second generation of Holocaust survivors (Giladi and Bell, 2012). A recent study which examined effects of political oppression in Lithuania found some interesting results, revealing that participants from families of political violence survivors had significantly higher psychological well-being (Kazlauskas and Zelviene, 2015) as compared to the families with no history of exposure to political violence. These findings were also supported by another study on intergenerational resilience in Lithuania (Mazulyte et al., 2014).

In line with the emerging ideas on intergenerational resilience, our study is grounded in the theory of salutogenesis proposed by Antonovsky (1987). The theory of salutogenesis focuses on a person’s ability to overcome difficulties in life, and is quite often applied in trauma research (Schnyder et al., 2000; Cassel and Suedfeld, 2006; van der Hal-van Raalte et al., 2008; Fossion et al., 2015). Sense of coherence, which is the key concept of salutogenesis, has three components: comprehensibility, manageability, and meaningfulness. The sense of coherence scale as a measurement instrument based on the theory of salutogenesis was proposed by Antonovsky (1987, 1993). Since then it has gained great popularity and has been translated to at least 49 languages, and has been used in 48 countries according to a recent review by Eriksson and Mittelmark (2017). Furthermore, sense of coherence measures have been applied while studying the effects of intergenerational trauma. Sense of coherence was found to have a mediating effect on depression and anxiety in the second generation of Holocaust survivors (Goldberg and Wiseman, 2014; Fossion et al., 2015). Within the theoretical framework of salutogenesis, we focus on intergenerational resilience (Shmotkin et al., 2011; Fletcher and Sarkar, 2013) rather than trauma transmission by analyzing the links between parents and their adult offspring sense of coherence in Lithuanian families with a history of political violence.

### Aim of the Study

The aim of this study was to explore post-traumatic stress in the adult offspring of Lithuanian survivors of political violence, and to evaluate the links between parental and their adult offspring’s sense of coherence in the families exposed to political violence during the oppressive communist regime in Lithuania. We hypothesized that parents and their offspring’s sense of coherence would be significantly associated.

## Materials and Methods

### Participants and Procedure

We extracted data for this study from a database built up during the research project “*Long-term Effects of Political Oppression in Lithuania*” which aimed at exploring long-term effects of political violence (1940–1991) in Lithuania, and was conducted by the Vilnius University Trauma Research Group in collaboration with the Lithuanian Genocide and Resistance Research Center (LGRRC) (Gailiene and Kazlauskas, 2005; Kazlauskas et al., 2012). The database includes demographical, trauma exposure, coping, and health data of a non-clinical sample of 1,404 survivors of political violence, and 211 adult offspring of survivors. The mean age of survivors of political oppression was 73.07 years at the time of research in the first wave of the study. All survivors had an official status of victims of political violence according to Lithuanian national laws (Republic of Lithuania Law on Legal Status of Victims of 1939–1990 Occupations, VIII-342, 1997). The first wave data was collected via face-to-face interviews (20%) and by using mailed questionnaires (80%) with a response rate of 80%. Second generation participants were recruited from the general population and by contacting the participants of the first wave of the study by phone, and asking to provide the contacts of their children for participation in the intergenerational study (second wave). After obtaining the consent of the participants of the study, questionnaires were mailed to the offspring of the survivors. The response rate in the second generation data collection was 48.3%. The procedures of data collection and study design have been described in detail in previous papers, and two doctoral dissertations (Gailiene and Kazlauskas, 2005; Kazlauskas, 2006; Kazlauskas et al., 2012; Vaskeliene, 2012).

We included 110 matched parent–child pairs from the same families in our analysis. The average age of the first generation survivors of political violence in this study was 73.22 (*SD* = 5.26) years, 60% (*n* = 66) women. The average age in the adult offspring group was 44.65 (*SD* = 6.55) years, 61.8% (*n* = 68) women. Sociodemographic characteristics of the sample are presented in **Table 1**.

**Table 1:** Sociodemographic characteristics of the sample.

	Parents (*n* = 110) *n* (%)	Adult offspring (*n* = 110) *n (%)*
Gender		
Men	44 (40.0)	42 (38.2)
Women	66 (60.0)	68 (61.8)
No of children		
0	0 (0.0)	11 (10.0)
1	20 (18.2)	24 (21.8)
2	54 (49.1)	57 (51.8)
≥3	36 (32.7)	18 (14.4)
Educational level		
Primary	39 (35.5)	1 (0.9)
Secondary	38 (34.5)	22 (20)
Professional college	17 (15.5)	32 (29.1)
University	16 (14.5)	55 (50.0)
Family status		
Married	81 (73.6)	83 (75.5)
Single	0 (0.0)	13 (11.8)
Widowed	27 (24.5)	3 (7.3)
Divorced	2 (1.4)	11 (10.0)


### Research Ethics

The study was conducted in accordance with national and international ethical regulations for psychological research. Lithuanian national psychological research ethical regulations do not require approval from the Institutional research board for the analysis of archival data, and additional written informed consent from participants is not required in such studies. Data was analyzed and presented in this publication according to the relevant national and international ethical guidelines, which also includes protection of the privacy of participants of the study. The dataset used for this paper was collected in the years 2002–2011 prior to the establishment of the Institutional research ethics boards for psychological research in Lithuania, which were founded in the year 2012. A written form of informed consent, describing the aims of the study and its procedures, was used in the process of data collection. Participants were informed about the possibility to withdraw from the study at any time.

### Measures

Life-time trauma exposure was measured with the Harvard Trauma Questionnaire (HTQ) (Mollica et al., 1992). The HTQ was developed for the assessment of post-traumatic stress and trauma exposure among torture survivors. The first part of the HTQ questionnaire includes a list of 23 stressful events, and was used for the assessment of both parental and adult offspring traumatic events. The participants were asked to indicate which traumatic events they experienced in their lifetime. Life-time trauma exposure was measured by the sum of life-time traumatic events indicated in the event list. Reliability and validity of the original version is adequate (test–retest *r* = 0.89, Cronbach’s alpha = 0.90) (Mollica et al., 1992). Lithuanian version of the HTQ was used in several studies in Lithuania (Domanskaité-Gota et al., 2009; Kazlauskas et al., 2012).

The Impact of Event-Scale Revised (IES-R) was used to measure post-traumatic stress disorder symptoms (Weiss and Marmar, 1997) in adult offspring of survivors. Participants were asked to select the most distressing experience from the list of potentially traumatic events, or add an additional event if needed before filling in responses to the IES-R items. Post-traumatic stress reactions were measured only among participants with at least one significant life-time traumatic event experience. The IES-R is a widely used 22-item self-report instrument based and DSM–IV (American Psychiatric Association, 1994) post-traumatic stress disorder symptoms and was proposed by Weiss and Marmar (1997), and includes three subscales measuring intrusion, avoidance and hyperarousal symptoms. We used a 5-point scoring system proposed by Weiss and Marmar (1997) with a scale ranging from 0 (= *Not at All*) to 4 (= *Extremely*) in our study. The total score for the IES-R is the mean of responses to all the items, and the score for the subscales are the means of the responses to items comprising the subscales. The Lithuanian version of the IES-R was used in several studies, including non-clinical (Kazlauskas et al., 2006; Kazlauskas and Zelviene, 2017) and clinical samples (Kazlauskas et al., 2017). We used a cut-off of IES-R > 1.5 for probable PTSD diagnosis (Creamer et al., 2003) which was used in previous Lithuanian studies (Kazlauskas and Zelviene, 2017; Kazlauskas et al., 2017). Good psychometric properties of the Lithuanian IES-R version was reported in previous studies with Cronbach’s alpha ranging from 0.93 to 0.96 for the IES-R total scale, and 0.82–0.88 for the IES-R subscales (Kazlauskas et al., 2006, 2017). Internal consistency of the total IES-R scale in this study among the second generation was high (α = 0.95).

A short 13-item Lithuanian version of the Sense of Coherence Scale (SOC-13) was used to measure parents’ and offspring’s sense of coherence in this study. Since the introduction of the Sense of Coherence Scale, several versions of this instrument have been developed (Eriksson and Lindström, 2005; Eriksson and Mittelmark, 2017) with the number of items ranging from 3 to 29. The SOC-13 scale is widely used in research as psychometric properties of this version is comparable with a full 29-item version of the Sense of Coherence Scale (Eriksson and Lindström, 2005). The SOC-13 includes items of the main aspects of sense of coherence: comprehensibility, manageability, and meaningfulness. Participants selected from a 5-point Likert type scale response to each of the items, ranging from 1 to 5. The total score of the SOC-13 was calculated by summing the responses to each of the items. The total score of SOC-13 ranged from 13 to 65 in this study, with a higher score indicating the stronger sense of coherence. The Lithuanian version of the SOC-13 demonstrated adequate psychometric properties in the previous studies with Cronbach’s alpha value of 0.85 among survivors of political violence (Kazlauskas, 2006). The SOC-13 Cronbach’s alpha in this study among survivors of political violence was 0.83, and in adult offspring sample was 0.81.

### Data Analysis

Analyses were conducted with IBM SPSS Statistics version 24. Due to incomplete responses to one or more items of the SOC-13, 10.9% (*n* = 12) parent, and 4.5% (*n* = 5) second generation sense of coherence data was missing. Missing data was handled by excluding the missing cases pairwise in each analysis.

Multiple regression analysis was used to predict the sense of coherence in the second generation. Variables significantly correlated with the offspring’s sense of coherence were included into the regression analysis as independent variables.

## Results

### Trauma Exposure in Survivors Group

All survivors of political violence reported exposure of at least one life-time traumatic event. The average number of life-time traumatic experiences in a sample of survivors of political violence was 6.94 (*SD* = 3.58). The majority of survivors of political violence experienced political imprisonment (66.4%; *n* = 73), a quarter of the survivors (25.2%; *n* = 28) experienced forced displacement to remote regions of Siberia, and 8.2% experienced other forms of political oppression. Political prisoners experienced an average of 6.4 (*SD* = 3.3; range 1–15) years of imprisonment for political reasons. About half of survivors (50.9%; *n* = 56) lost their family members because of political violence. The majority of survivors (88.2%; *n* = 97) reported they could not achieve their personal or professional goals in life because of political oppression.

### Trauma Exposure and Traumatic Stress in Adult Offspring

In the adult offspring sample 88.2% (*n* = 97) of participants reported exposure to at least one life-time traumatic event. The average number of life-time traumatic events in the adult offspring group was 3.88 (*SD* = 3.34). The most prevalent traumatic events in the sample were the loss of a family member (55.5%, *n* = 61), robbery (35.5%, *n* = 39), car accident (30.9%, *n* = 34), and serious disease (29.1%, *n* = 32).

Post-traumatic stress reactions were assessed using the IES-R only in the subsample of the participants who reported at least one life-time traumatic event. The total score for the IES-R in the adult offspring group with reported exposure to a traumatic event was 0.85 (*SD* = 0.84), and for the IES-R subscales ranged from 0.76 to 0.98 (**Table 2**). Probable PTSD was identified in 32 (29.1%) of the adult offspring group participants based on a cut-off of >1.5 for the total IES-R score. There were no significant associations between post-traumatic stress symptoms and the number of life-time traumatic events in the offspring group.

**Table 2:** Means, standard deviations, and correlations between trauma exposure, sense of coherence and post-traumatic stress among adult offspring.

Measure	*M*	*SD*	1	2	3	3.1	3.2
1 Life-time traumatic events	3.88	3.37					
2 Sense of coherence	46.70	6.07	-0.09				
3 IES-R Total scale	0.85	0.84	0.08	-0.36^∗∗∗^			
3.1 IES-R Intrusion	0.95	1.00	0.14	-0.35^∗∗^	0.95^∗∗∗^		
3.2 IES-R Avoidance	0.80	0.94	0.04	-0.44^∗∗∗^	0.94^∗∗∗^	0.87^∗∗∗^	
3.3 IES-R Hyperarousal	0.76	0.78	0.05	-0.20	0.89^∗∗∗^	0.74^∗∗∗^	0.74^∗∗∗^


*IES-R, Impact of Event Scale-Revised. ^∗∗^p < 0.01; ^∗∗∗^p < 0.001*.

### Sense of Coherence

The average SOC-13 score among the first generation of survivors was 45.47 (*SD* = 8.15). In the adult offspring group the average SOC-13 score was 46.70 (*SD* = 6.07). Significant correlation was found between parental and adult offspring’s sense of coherence (*r* = 0.37, *p* < 0.001).

We found no significant associations between parental life-time traumatic experiences and other measures in our study. Parental sense of coherence was not associated with the number of survivors’ life-time traumatic events (*r* = -0.10, *p* = 0.309). No significant correlation was found between parental sense of coherence and offspring post-traumatic stress reactions (*r* = -0.04, *p* = 0.693). The parental number of life-time traumatic events was not associated with offspring’s sense of coherence and post-traumatic stress.

Post-traumatic stress reactions significantly correlated with a sense of coherence in the adult offspring group (**Table 2**), except hyperarousal, which was not significantly correlated with the SOC-13. The sense of coherence in the adult offspring subsample with probable PTSD was significantly lower (*M* = 43.61; *SD* = 6.11) in comparison to the adult offspring subsample with no PTSD (*M* = 47.75; *SD* = 5.59) [*t*(91) = 3.19, *p* < 0.01]. The effect of probable PTSD on sense of coherence was medium (between group Cohen’s *d* = 0.71). There was no significant associations between offspring’s sense of coherence and age (*r* = -0.07, *p* = 0.470). We also did not find significant gender effects on offspring’s sense of coherence [*t*(103) = 0.60, *p* = 0.550].

We included parental sense of coherence and adult offspring PTSD reactions, measured with the total IES-R score in a regression analysis for the prediction of adult offspring’s sense of coherence. The regression model explained 23.7% of the variance, and both parental sense of coherence and child post-traumatic stress reactions predicted the sense of coherence of adult offspring significantly (**Table 3**).

**Table 3:** Multiple regression analysis predicting sense of coherence in adult offspring.

Dependent variable	Predictors	*B*	*SE*	β	*F*	*R*^2^
Sense of coherence in adult offspring	Parental sense of coherence	0.24	0.08	0.32^∗∗^	12.15^∗∗∗^	0.24
	Adult offspring post-traumatic stress symptoms	-2.61	0.74	-0.35^∗∗^		
	Constant	37.71	3.54			


^∗∗^*p* < *0.01*; ^∗∗∗^*p* < *0.001*.

## Discussion

This is one of the first studies that explored the links between parental and second-generation sense of coherence in the families of political violence survivors. We found a significant association between survivors of political violence and their adult offspring’s sense of coherence. We also found high levels of post-traumatic stress symptoms in the second generation with 29% of adult offspring having a probable PTSD based on self-report measures. Prevalence of probable PTSD in the second generation was higher in comparison to prevalence of PTSD in Lithuania, which range from 2 to 7% of the population as reported in a review of PTSD studies in the Baltic States (Kazlauskas and Zelviene, 2016). Moreover, probable PTSD rates in this study were significantly higher in comparison to the 8% prevalence of potential PTSD found in a recent study of 778 participants from the general population of Lithuania, using the same IES-R cut-off score (Kazlauskas and Zelviene, 2017).

Our sample was not a representative sample of the Lithuanian second generation of political violence survivors, and there is possibility that offspring with higher levels of psychopathology were more interested in participating in this study. However, we identified an increased vulnerability of adult offspring of survivors of political violence. This increased vulnerability could be related to higher risks for the offspring of survivors in their childhood. All children of survivors in our study were born during the period of Soviet political oppression. Second generation participants were potentially exposed to high risk factors in childhood which could have contributed to intergenerational effects (Serbin and Karp, 2004) due to insecurity in families (Vaskeliene, 2012). The average number of traumatic events in the second generation of this study (*M* = 3.9) was higher in comparison to the general population of Lithuania (*M* = 1.6) (Kazlauskas and Zelviene, 2017), and this has possibly also contributed to increased probable PSTD rates in our sample. However, association between the number of life-time traumatic events and PTSD symptoms in the second generation was non-significant.

Furthermore, psychological support and treatment programs for survivors are very important, and successful rehabilitation programs for Holocaust survivors have been proposed many years ago (Kellermann, 2001). These services and programs were not available to Lithuanian survivors for decades during political oppression until 1990s, when Lithuania regained its freedom (Kazlauskas et al., 2016). In addition, there is also a growing apprehension of the abuse of psychiatry in the context of political oppression around the world, including the period of Soviet occupation (van Voren, 2010; Buoli and Giannuli, 2017). It is estimated that almost one third of political prisoners were abused by the psychiatric system in the former communist Soviet Union (Buoli and Giannuli, 2017). The prevalent negative attitudes toward psychiatry which were perceived as dangerous by the family members of survivors of political oppression might have resulted in the avoidance of mental health services even when they were needed.

We found a negative correlation between the sense of coherence and post-traumatic stress symptoms in the second generation. This finding is in line with previous findings (e.g., van der Hal-van Raalte et al., 2008), and is rather obvious taking into account the theoretical framework of salutogenesis (Antonovsky, 1987; Mittelmark et al., 2017). However, the most interesting finding of this study was a significant association of a sense of coherence between parents and adult offspring in the families of survivors. To a certain extent, a stronger sense of coherence is also related with a higher resiliency of a person, and the capacity to confront the stressor with more resistance. In this light, our findings might indicate that parental resilience is associated with child resilience. The idea that sense of coherence is related with resilience was discussed earlier in other studies (Almedom, 2005), and it was hypothesized that at least some parts of sense of coherence are related with resilience (Fossion et al., 2015).

Surprisingly, little is known about the relationship between parental and offspring’s sense of coherence from empirical studies. Studies mostly analyze how a sense of coherence is associated with health in a sample of survivors of trauma, or the second generation. There is some evidence that parental sense of coherence might be associated with the health of children with diabetes (Goldberg and Wiseman, 2014) indicating that there are possible links among parents and their offspring’s health. However, there are no such studies in the context of intergenerational trauma to our knowledge. Because of the lack of such studies we can only hypothesize how sense of coherence might be transmitted to offspring in families affected by political violence. It is possible that survivors with a higher sense of coherence had less post-traumatic stress reactions, their parental style was more adaptive and thus it resulted in a stronger sense of coherence in the second generation and presumably higher resilience (Schofield et al., 2014). A positive parenting style and a stronger sense of coherence related to active coping might be important factors for intergenerational resilience (Rowland-Klein and Dunlop, 1998). In the context of growing evidence on genetic and epigenetic factors of trauma transmission (Bowers and Yehuda, 2016), we could assume that parental behaviors can prevent triggering the stress-response systems that impair mental health in offspring.

There are several implications of our findings for clinical practice. Clinicians should be aware of the role of the traumatic history of the family on the second generation of survivors in clinical setting. The intergenerational transmission of resilience indicates the need to focus not only on mental disorders, but also on resilience in treatment. Several attempts to develop an intervention based on the salutogenic approach showed promising results (Langeland and Vinje, 2017). Salutogenic therapy aims to increase coping and a sense of coherence by focusing on the internal resources of a client (Langeland and Vinje, 2017). Furthermore, mental health professionals could seek to facilitate development of positive self-identity (Antonovsky, 1987) among their clients to increase resilience.

While we have found interesting results, there are limitations of this study that need to be addressed. Firstly, the sampling of this study was based on referrals from parents for inclusion of their offspring in a study. However, even taking into account the bias in sampling we could identify the increased vulnerability of the second generation of the families with a background of political oppression. Identification of higher probable PTSD rates than the general population also raises the question recently brought up by Danieli et al. (2016) in response to recent debates on trauma transmission. Rather than questioning if there are any intergenerational effects, we should rather focus on identifying the characteristics of individuals who are affected by parental trauma, which not only supports the need for future intergenerational studies in Lithuania, but also in other populations. Secondly, we included only survivors of political violence who were still alive after almost 50 years of political oppression while many died during the oppression. Health and a sense of coherence of survivors might be higher than all the other victims in this group. There is evidence from mortality studies that strong sense of coherence contributes to lower mortality (Surtees et al., 2003). With a highly selective sample of elderly survivors it is possible that intergenerational transition of resilience was more possible, as survivors are probably more resilient. Thirdly, we speculate about a sense of coherence in terms of resilience in this paper. There are scales for measuring resilience available (e.g., Connor and Davidson, 2003). Further studies using resilience measures could provide more insights into intergenerational resilience. Finally, there are study design related limitations as well. We did not include a comparison group in this study, and our study design cannot answer if parent–child associations of sense of coherence are found only among the members of a highly traumatized population. We were not able to analyze intergenerational trauma transition in this study because of the lack of the parental post-traumatic stress symptoms data in our study. In addition, this was not a longitudinal study, and further longitudinal studies could provide more insights on the development of sense of coherence in the offspring of traumatized parents.

Despite these limitations, we found promising results supporting the concept of intergenerational resilience transmission. We managed to analyze matched pairs of survivors of prolonged traumatization and their adult offspring in a new sample with a new approach using a sense of coherence measure. Our study brings up questions regarding resilience and positive aspects of intergenerational trauma in families. Further studies are needed to test the findings of this study in other populations.

## Ethics Statement

This study was carried out in accordance with the Lithuanian ethical regulations for psychological research. All participants of this study gave an informed consent for participation in the study.

## Author Contributions

EK has planned the study, proposed the idea for the manuscript, performed statistical data analysis, and wrote the first draft of the manuscript. DG significantly contributed to the planning of the study, reviewed the draft of the manuscript, and made significant contribution to it. IV contributed to data collection, reviewed the first draft of the manuscript, and made significant contribution to it. MS-K reviewed the first draft of the manuscript, and made significant contribution to it.

## Conflict of Interest Statement

The authors declare that the research was conducted in the absence of any commercial or financial relationships that could be construed as a potential conflict of interest.
